# Heating and Ventilation

**Published:** 1905-07-01

**Authors:** 


### HEATING AND VENTILATION.

### ANNUAL MEETING OF THE INSTITUTE OF HEATING AND VENTILATING ENGINEERS AT BRISTOL.

This is one of the younger engineering institutes. It has been established to deal especially with the very important problems involved in heating and ventilating, and it is composed mainly of men who are engaged in practical work in those branches of engineering. It is doing very good and very solid work. At the annual meeting two papers were read, one by Professor Barrett, of Dublin, who is an honorary member of the Institute, "On the Heating of Buildings and on Dust Deposition," and the other by Mr. Row, the managing director of Royles, Limited, the well-known heating engineers, "On Steam Economy in Connection with the Heating Trade." Professor Barrett's paper struck us as singularly weak, from the point of view from which it should have been strong. A professor of physics can hardly be expected to have an intimate acquaintance with the numberless little points which go to make up what is called practical experience in heating and ventilating, but he should be able to interpret the experience of the men who are engaged in the work; this, however, is what Professor Barrett appeared to be unable to do. He stated a few facts, such as that when a globe is placed over an ordinary gas-burner, the gas being consumed inside the globe, instead of freely in air, a better heating result is obtained; but he was unable to explain it. The professor had made measurements of the temperature, but that was as far as he had gone. It never appears to have occurred to him that the result was due to more perfect combustion. Similarly he stated the fact that dust is deposited on cold surfaces, but again beyond a list of names of eminent men who had conducted some experiments in connection with the matter and who spoke at the meeting, he was unable to clear up the mystery of why the deposit occurred. Practical men had very little to say, and had discovered a remedy and had ideas of the cause. The "Plenum" system he damned with faint praise, and again laid himself open to the obvious criticism that he had not really studied the question at all. He appeared to think that air-locks were necessary for each ward, and that it was impossible to open windows where you had the system, and to be very much terrified at the thought that if something went wrong with the apparatus you would have trouble. This is all terribly weak, as it is neither the impartial summing up of the judge nor the vigorous attack of the advocate of opposing systems. It is merely the half-hearted essay of a man who knows very little about the matter, but who fancies the system is bad because he has been told so. The real question, What is there behind the "Plenum" system that makes men feel, as they declare, a sensation of staleness, when constantly living in the atmosphere, he did not attempt to deal with. One point he mentioned in connection with the "Plenum" system that has not been mentioned before, and that is, the possibility of germs being deposited with the dust, in the exit ducts. We should like to see this taken up in the hospitals where the system is in use. The "Plenum" representatives vigorously questioned all the professor's adverse conclusions, including that referring to dust being deposited in the exit ducts.

The other paper was full of useful, debatable matter. It gave the experience of a man actively engaged in fitting heating apparatus, and some of its conclusions were traversed by other men who were also engaged in the same work. One important point that was brought out in the discussion was the fact that it is not so economical to heat by high pressure steam, as by low pressure, and the lower the pressure the greater the economy, because the heating effect is due to the condensation of the steam, and the latent heat of high pressure steam is less than that of low pressure. This is the kind of question we should have expected Professor Barrett to have taken hold of, and explained, but it was a practical man who brought the matter out, in a properly scientific manner. Similarly in the professor's own paper, he referred to the question of heating water by injecting steam directly into it, saying that he did not see why it should not be applied. Any heat engineer's apprentice could have told him what the difficulty was in applying that method. The professor never brought out the important fact that while the proportion of the total heat in the steam that can be delivered to the water to be heated in the ordinary way with calorifiers is small, the whole of the heat must pass into the water with the injection system. It was a practical man who brought this fact out. Mr. Row's paper contained several valuable tables, valuable from the fact that though, as was pointed out, there were inaccuracies in them, they represented the results of measurements under practical conditions. One table struck us as of considerable value, in which the author of the paper gave the results of a series of experiments on the heating of air by passing it over a steam heating apparatus. The interest, to our mind, lies in the fact that only a small percentage of the total heat used in the heating apparatus was delivered to the air; when the air travelled at 2,000 cubic feet per minute the highest we made out to be about 7 per cent., and it was increased from about 5 per cent. to 7 per cent. when it travelled at 5,000 cubic feet per minute. We should like to see the experiments carried farther. There will be a critical speed at which no further increase is obtainable, and that should be the speed at which the air passes through the heating apparatus, reduced afterwards if necessary.

Among the works, etc, visited by the Institute, we may mention the public baths of the Bristol Corporation, where the working man can have what is practically a Turkish bath, he acting as his own shampooer, for fourpence. swimming bath also presents some very interesting fea[illegible] the water, in addition to being changed twice weekly ( he bath being thoroughly cleaned each time), is constantly drawn off in small quantities, aerated, filtered, and passed back into the bath warmed again. The bath is warmed by a steam injector. The water, on the occasion of our visit, was beautifully clear.

